# Unveiling the pathogenesis and therapeutic approaches for diabetic nephropathy: insights from panvascular diseases

**DOI:** 10.3389/fendo.2024.1368481

**Published:** 2024-02-22

**Authors:** Xiaoqian Zhang, Jiale Zhang, Yan Ren, Ranran Sun, Xu Zhai

**Affiliations:** ^1^ Department of Nephrology, Beijing Hospital of Integrated Traditional Chinese and Western Medicine, Beijing, China; ^2^ Institute of Basic Theory for Chinese Medicine, China Academy of Chinese Medical Sciences, Beijing, China; ^3^ Xiyuan Hospital of China Academy of Chinese Medical Sciences, Beijing, China; ^4^ Dongfang Hospital, Beijing University of Chinese Medicine, Beijing, China; ^5^ Wangjing Hospital, China Academy of Chinese Medical Sciences, Beijing, China

**Keywords:** diabetic nephropathy, panvascular disease, endothelial dysfunction, renin-angiotensin system, novel antidiabetic drugs, individualized therapy

## Abstract

Diabetic nephropathy (DN) represents a significant microvascular complication in diabetes, entailing intricate molecular pathways and mechanisms associated with cardiorenal vascular diseases. Prolonged hyperglycemia induces renal endothelial dysfunction and damage via metabolic abnormalities, inflammation, and oxidative stress, thereby compromising hemodynamics. Concurrently, fibrotic and sclerotic alterations exacerbate glomerular and tubular injuries. At a macro level, reciprocal communication between the renal microvasculature and systemic circulation establishes a pernicious cycle propelling disease progression. The current management approach emphasizes rigorous control of glycemic levels and blood pressure, with renin-angiotensin system blockade conferring renoprotection. Novel antidiabetic agents exhibit renoprotective effects, potentially mediated through endothelial modulation. Nonetheless, emerging therapies present novel avenues for enhancing patient outcomes and alleviating the disease burden. A precision-based approach, coupled with a comprehensive strategy addressing global vascular risk, will be pivotal in mitigating the cardiorenal burden associated with diabetes.

## Introduction

According to the International Diabetes Federation’s report, over 530 million people worldwide have diabetes (1). About one-third of diabetic patients develop diabetic nephropathy (DN) after the incubation period, which may last several years (2). Diabetic nephropathy, as a common complication of diabetes, has drawn widespread attention globally. With the increasing number of diabetes patients and lifestyle changes, DN is rising. DN is characterized by structural and functional kidney damage due to prolonged high blood glucose levels, resulting in a gradual decline in glomerular filtration rate, proteinuria, and progressive renal function impairment (3). It is one of the leading causes of chronic kidney disease (CKD) and a major contributing factor to end-stage renal disease (ESRD) (4).

Additionally, diabetes patients often risk panvascular diseases (5), which involve the entire vascular system, including atherosclerosis, cardiovascular diseases, and cerebrovascular disorders. Due to alterations in the vascular wall structure and function in diabetes patients, they are more prone to developing atherosclerosis. Factors such as endothelial dysfunction, inflammation, oxidative stress, and platelet activation play important roles in promoting the progression of atherosclerosis in diabetes patients (6). The treatment strategies for DN and panvascular diseases include controlling blood glucose levels, managing blood pressure, restricting protein intake, and using renal protective agents. Furthermore, addressing atherosclerosis and cardiovascular diseases is crucial, involving lipid management, antiplatelet therapy, and cardiovascular protection measures (7, 8).

DN often has varying degrees of systemic vascular damage, making DN’s development more complex and severe. On the one hand, panvascular diseases accelerate the progression of DN, leading to a further decline in glomerular filtration rate, worsening proteinuria, and impaired renal function. On the other hand, DN is an independent risk factor for panvascular diseases, increasing the risk of cardiovascular events and all-cause mortality. The connection between DN and panvascular diseases is significant for preventing, diagnosing, and treating these conditions. While investigating the relationship between DN and panvascular diseases, several key questions deserve further exploration. Firstly, it is important to understand the common pathological mechanisms between DN and panvascular diseases. Factors such as high blood glucose, inflammation, and oxidative stress play crucial roles in both diseases. Secondly, it is necessary to explore how panvascular diseases affect the development and prognosis of DN. Further understanding the interplay between the two conditions can aid in developing more precise treatment strategies and preventive measures.

While further investigating the connection between DN and panvascular diseases, this perspective article addresses the following key questions:

### Common pathological mechanisms

High blood glucose, inflammation, and oxidative stress are important in DN and panvascular diseases. It is crucial to delve into these shared pathological mechanisms and explore how they interact to exacerbate disease progression.

### Impact of panvascular diseases on DN

How do panvascular diseases affect the development and prognosis of DN? Does it increase the risk of cardiovascular events and all-cause mortality? Exploring these key questions can enhance our understanding of the overall risk in DN patients and guide relevant interventions.

### Treatment strategies

More precise treatment strategies can be developed based on a thorough understanding of the connection between DN and panvascular diseases. Identifying common treatment targets and developing targeted therapeutic drugs may improve patients’ prognosis and quality of life.

## Molecular pathogenesis of DN: insights from panvascular

DN is one of the most common vascular complications of diabetes (9). In the early stages, abnormalities in the glomerular filtration membrane arise, characterized by thickening of the glomerular basement membrane and proliferation of mesangial cells, resulting in altered glomerular filtration rate (GFR). Concurrently, endothelial cell injury within the glomerulus and dysfunction of tubular epithelial cells manifest. The progression of disease leads to glomerulosclerosis, which is marked by substantial glomerular injury and proteinuria (10). Glomerulosclerosis and mesangial proliferation further exacerbate the decline in GFR. The advancement of DN may also induce interstitial fibrosis and tubular atrophy, impairing tubular function, including urine concentration ability and acid-base balance. In the late stages of DN, a gradual reduction in GFR ensues, intensifying glomerular dysfunction and aggravating glomerulosclerosis. Consequently, interstitial fibrosis, arterial sclerosis, and renal artery lesions may manifest in the kidney (11). The pathogenesis involves multiple molecular and cellular abnormalities. High blood glucose levels represent a primary risk factor for DN development in diabetic patients. Direct injury of renal vascular endothelial cells and podocytes by hyperglycemia plays a pivotal role in DN progression (12). Inflammation and oxidative stress significantly affect DN by activating inflammatory signaling pathways and induction of cellular apoptosis, thereby contributing to renal pathological changes (13). Additionally, aberrant activation of the renal renin-angiotensin system (RAS) and other signaling cascades promotes DN advancement (14). At the cellular level, prolonged hyperglycemic exposure elicits mitochondrial dysfunction, cellular metabolic derangements and oxidative stress in renal cells. This triggers the emission of damage-associated molecular patterns that stimulate innate immune cascades, leading to glomerular and tubulointerstitial inflammation (15). Pro-inflammatory cytokines such as interleukin-1β (IL-1β) and tumor necrosis factor-α (TNF-α) exacerbate podocyte and endothelial injuries via multiple mechanisms involving caspases, SMAD pathways and Rho-associated protein kinase. Concomitantly, hyperglycemia-induced advanced glycation end products (AGEs) formation and their engagement with renal receptors for AGEs amplify inflammation and fibrosis through diverse intracellular signaling molecules including protein kinase C, transforming growth factor-β and nuclear factor-κB (NF-κB) (16, 17). The intrarenal RAS is concurrently activated through Ang II and further propagates oxidative stress, inflammation, and extracellular matrix accumulation in DN progression via hemodynamic and non-hemodynamic effects (18, 19). These multi-factorial and inter-related pathophysiological processes synergistically promote renal structural and functional impairments characteristic of DN.

Here, we discuss the key pathophysiological processes at the cellular and molecular levels based on current evidence:

## Formation of advanced glycation end products and renal injury

The formation of advanced glycation end products (AGEs) is a complex process in hyperglycemia. Under hyperglycemia, non-enzymatic glycation of proteins/lipids leads to excessive AGEs accumulation, a hallmark of diabetic dysmetabolism (20). The binding of AGEs to receptors such as RAGE on renal endothelial cells activates pro-oxidative and pro-inflammatory pathways including NADPH oxidase, MAPK and NF-κB, inducing oxidative stress and inflammation (21, 22). Several studies have demonstrated the detrimental effects of AGEs and RAGE activation in the pathogenesis of diabetic nephropathy (23). For example, blocking RAGE activation has been shown to alleviate renal injury and reduce inflammation in experimental models of diabetes (24). Additionally, inhibition of the NADPH oxidase system, which is upregulated by RAGE signaling, has been found to attenuate oxidative stress and improve renal function in DN (25). Furthermore, inhibition of MAPK and NF-κB pathways has shown promising results in reducing inflammation and fibrosis in diabetic kidney disease (26, 27).

## Increased oxidative stress and inflammation

Mitochondrial dysfunction driven by persistent hyperglycemia plays a pivotal role in diabetes-induced oxidative stress and kidney inflammation. Sustained hyperglycemia leads to impaired ATP generation in renal cells by disrupting mitochondrial function (28). It has been shown that hyperglycemia decreases mitochondrial electron transport chain complexes I and IV activities, compromising the balance between superoxide production and dismutation in renal cells (29, 30). This triggers an overproduction of reactive oxygen species (ROS), such as superoxide anions.

Concurrently, hyperglycemia activates inflammatory pathways by upregulating proinflammatory mediators and accumulating immune cells in the kidneys. Hyperglycemia stimulates NF-κB and MAPK signaling pathways, leading to increased expression of cytokines, including IL-1β, TNF-α, and MCP-1 (31, 32). These inflammatory cytokines amplify local inflammation by recruiting monocytes that differentiate into resident proinflammatory M1 macrophages in the renal interstitium (33). Renal tubular epithelial cells also secrete chemokines that perpetuate inflammation (34).

Elevated ROS production results in oxidative damage to lipids and proteins, impairing cell membrane integrity and function (35). Inflammatory cytokines can activate hormonal systems to induce renal tubular cell apoptosis (36). Abnormal extracellular matrix remodeling involving increased collagen deposition and osteopontin contributes to renal interstitial fibrosis (37). In summary, mitochondrial dysfunction-induced oxidative stress and inflammation form a vicious cycle that cooperatively drives the pathogenesis of diabetic kidney disease.

## Endothelial dysfunction and increased permeability

Hyperglycemia elicits direct damage to endothelial cells lining the renal vasculature and glomeruli, manifesting as impaired endothelium-dependent vasorelaxation (38, 39), weakened homeostatic control of vascular tone and permeability (40, 41), and deregulated integrity of the glomerular filtration barrier (42). Chronic hyperglycemic conditions trigger excessive production of reactive oxygen species and proinflammatory signaling, which promote endothelial activation and a proinflammatory phenotype.

**Figure 1:**
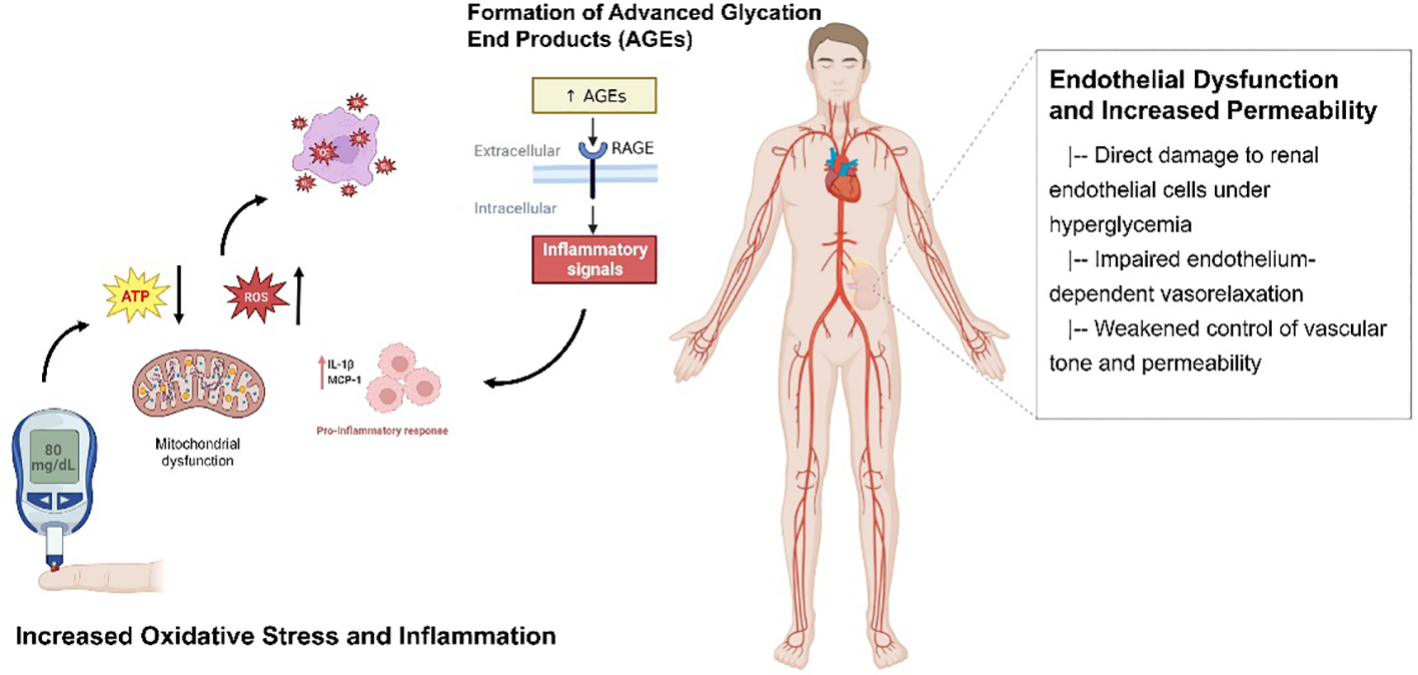
The relevant mechanisms and possible approaches for DN. The figure illustrates the critical mechanisms involved in renal injury in diabetes mellitus. Increased oxidative stress and inflammation, mitochondrial dysfunction driven by persistent hyperglycemia, and the formation of advanced glycation end products (AGEs) contribute to renal damage. Additionally, endothelial dysfunction and increased permeability further exacerbate the pathogenesis of diabetic nephropathy.

Activated endothelial cells exhibit disturbances in barrier function, increasing vascular permeability at the glomerular (43) and tubular (44) vascular beds. This allows plasma proteins such as albumin to leak into the urine, presenting clinically as proteinuria - a hallmark of DN (45). At the molecular level, hyperglycemia enhances endothelial expression of adhesion molecules that recruit leukocytes (46), decreasing the synthesis of antiproteinuric factors (47). The combined effects of direct hyperglycemic toxicity, oxidative stress and chronic low-grade inflammation thus converge to induce endothelial dysfunction in the renal vasculature. **Figure 1** shows the relevant mechanisms and possible approaches.

## The common relationship between DN and panvascular disease

The common pathological processes of DN and panvascular disease include vascular injury, inflammatory response, oxidative stress and fibrosis (48). At the same time, patients with DN are at risk for systemic vasculopathy. It has been found that diabetic patients are often accompanied by systemic vascular lesions such as abnormal vascular function, atherosclerosis, and platelet activation (49). These vasculopathies are somewhat similar to the development of DN, in which vascular endothelial cell injury, inflammatory response and oxidative stress are common pathological features (50, 51). Thus, DN and panvascular disease may share common molecular mechanisms that further exacerbate the development of DN.

Inflammatory response and oxidative stress are important common links. The inflammatory response is activated in both diseases, leading to increased production of inflammatory cytokines (52). These cytokines include tumor necrosis factor-α (TNF-α) (53), interleukin-1β (IL-1β) (54), and interleukin-6 (IL-6) (55), among others, which are involved in the inflammatory response of the vascular wall and damage to vascular endothelial cells (56). The increase in inflammatory cytokines further activates the NF-κB signaling pathway, promoting the inflammatory response’s continuation.

Under a hyperglycemic state, excessive glucose metabolism generates many oxygen free radicals, disrupting the intracellular redox balance. In addition, oxidative stress in diabetic patients can be caused by mitochondrial dysfunction and accumulation of glycosylation end products. Excessive production of oxygen free radicals and imbalance of the antioxidant system leads to increased intracellular oxidative stress, further damaging renal and vascular cells (57).

In addition to inflammatory responses and oxidative stress, several important molecular pathways and signaling molecules are involved in developing DN and panvascular disease (58). For example, transforming growth factor-β (TGF-β) (59) is important in both diseases. TGF-β is involved in thickening and fibrosis of the glomerular basement membrane, disrupting the glomerular filtration barrier. In addition, angiotensin II (Ang II) has been implicated as a co-regulatory molecule in DN and panvascular disease, exacerbating the progression of both diseases through mechanisms that promote vasoconstriction and increase inflammatory responses and oxidative stress (60, 61).

A close interaction exists between renal vascular abnormalities in DN and systemic vascular complications. Persistent hyperglycemia can exacerbate systemic vascular injuries through renal endothelial dysfunction, inflammation and the spill over of vascular reactive species and cytokines. Meanwhile, panvascular diseases such as atherosclerosis can directly impair renal hemodynamics by narrowing intrarenal arterioles and compromising renal blood supply and pressure profiles, undermining glomerular filtration function. Furthermore, advanced glycation end products and platelet hyperreactivity may precipitate thrombotic occlusions obstructing renal microcirculation, augmenting ischemic injuries to the glomeruli. These reciprocal effects between renal and systemic vessels collectively form a vicious cycle that reinforces the progression of DN and panvascular comorbidities in diabetes.

## Treatment strategies for DN

Tight glycemic and blood pressure control are recognized as cornerstone therapies for DN (62). Mounting evidence suggests that stringent control of hyperglycemia effectively mitigates renal complications (63). Clinical studies (64, 65) have validated that achieving near-normal glycemia through a combinatorial pharmacotherapy approach, dietary modification and exercise substantially delays DN progression and lowers risks of renal adverse outcomes. Hypertension is also a key driver of DN pathogenesis (66, 67). Effectively managing blood pressure alleviates hemodynamic overload on the kidneys and hampers disease progression, as corroborated by numerous trials (68, 69). Given individual variations in disease severity and responses, glycemic and blood pressure control treatment targets should be personalized. Close monitoring with timely adjustments is pivotal to minimizing clinical deterioration and organ damage over the long term. With a treatment paradigm centered around intensive management of the two metabolic abnormalities, multidisciplinary care integrating medical, lifestyle and educational elements can help optimize renal protection in this high-risk population. Achieving recommended targets demands relentless efforts from patients and healthcare providers alike.

Chronic inflammation and oxidative stress play pivotal roles in the pathogenesis and progression of DN. Targeting these pathogenic processes represents a promising therapeutic strategy. Preclinical evidence (70) suggests anti-inflammatory interventions, including non-steroidal anti-inflammatory drugs, glucocorticoids, and anti-cytokine therapies, may attenuate renal inflammation and fibrosis in DN to a certain extent (71). Based on the 2024 latest research finding (72) by the American Diabetes Association Professional Practice Committee, cardiovascular event risk reduction in DN patients is recommended through finerenone, a non-steroidal selective mineralocorticoid receptor antagonist. This medication has been clinically proven to reduce cardiovascular events and the progression of chronic kidney disease (73). Recently, guidelines (74–77) reflect growing recognition of finerenone’s clinical benefits and increasingly emphasize the need for earlier intervention strategies that concurrently target cardiorenal protection in patients with coexisting diabetes, kidney disease and cardiovascular illness.

Similarly, antioxidative agents such as vitamins E and C and glutathione have demonstrated renoprotective effects by ameliorating oxidative insults and preserving renal and vascular cellular integrity from radical-mediated damage (78–80). Pentoxifylline (PTF) is a methylxanthine derivative and a phosphodiesterase inhibitor that can inhibit the production of pro-inflammatory cytokines such as tumor necrosis factor-alpha and interleukin-1 beta. It reduces inflammation and has a significant therapeutic effect on DN (81). PTF exerts its anti-proteinuric effects through various mechanisms, including improving renal microcirculation, inhibiting oxidative stress, and reducing collagen deposition (82). Clinical studies (83, 84) have shown that PTF can significantly decrease proteinuria in DN patients and improve CRP and TNF-α. Therefore, PTF has become an important adjunctive therapy for DN. However, larger and longer clinical outcome trials are still warranted to definitively establish the efficacy and safety profile of anti-inflammatory and antioxidative therapies in DN management. Unresolved questions around optimal drug selection, dosing regimen, duration of intervention and long-term benefits need to be addressed to inform clinical recommendations. Nonetheless, given their mechanistic rationale targeting the underlying chronic pro-oxidative and pro-inflammatory milieu driving DN progression, further exploring these therapeutic avenues through well-designed studies remains an active area of research interest. Combinatorial regimens harnessing multiple protective mechanisms may also hold promise.

The renin-angiotensin-aldosterone system (RAAS) axis plays a pivotal role in the pathogenesis of DN. RAAS blockade with ACE inhibitors (ACEi) and angiotensin receptor blockers (ARB) are well-established therapeutic interventions for DN. Angiotensin II receptor antagonist losartan can effectively reduce the progression of nephropathy in patients with type 2 diabetes. Compared to conventional antihypertensive treatment, losartan can lower the risk of renal function deterioration, end-stage renal disease, and death (85). These agents retard DN progression by lowering intraglomerular pressure and mitigating renal inflammation. However, their use requires close monitoring due to potential adverse effects such as worsening kidney function and hyperkalemia in some patients. Sodium-glucose cotransporter 2 (SGLT2) inhibitors are emerging antidiabetic agents. They promote glucosuria and lower blood glucose levels by inhibiting SGLT2-mediated glucose reabsorption in proximal tubules (86). Recent clinical trials (87, 88) demonstrated their renoprotective benefits in DN, including reductions in proteinuria and slower eGFR decline. They also confer cardiovascular protection.

Glucagon-like peptide-1 (GLP-1) receptor agonists stimulate insulin secretion and inhibit intestinal glucose absorption. Emerging evidence (89, 90) indicates their treatment may confer renal benefits in DN, such as decreasing proteinuria, improving glomerular filtration rates and attenuating fibrosis.

In summary, while RAAS blockade forms the mainstay of pharmacotherapy for DN, SGLT2 inhibitors and GLP-1RAs hold promise as adjunctive therapies given their additional reno- and cardio-protective effects observed in recent landmark outcome trials. Their integration into routine clinical care warrants further investigation.

## Emerging treatments and future perspectives

### Epigenetics and metabolic memory

Individualized therapy and precision medicine also hold great potential in managing DN. Recent findings (91, 92) have elucidated the roles of epigenetics and metabolic memory in linking genetic and environmental risk factors. Mechanisms, including DNA methylation, histone modification, and non-coding RNA regulation, are involved, promoting the development of metabolic memory and leading to a poor prognosis in DN (93, 94). Long non-coding RNAs (lncRNAs) help regulate these epigenetic changes and drive gene expression changes in DN pathogenesis. While our mechanistic understanding has progressed, effective therapies still need to be improved. A precision medicine approach integrating multi-omics profiling with clinical characteristics holds promise to precisely tailor individualized treatment for DN. Targeting disease-relevant lncRNAs may uncover new opportunities for genomic medicine to treat DN.

### NETosis and neutrophil extracellular traps

NETosis, the formation of neutrophil extracellular traps (NETs), plays a significant role in the pathogenesis of DN (95). While inflammation and oxidative stress are known contributors to DN, the involvement of neutrophils has been largely overlooked. Elevated glucose levels increase PKC activity, NADPH-oxidase overstimulation, and oxidative burst, irrespective of diabetes type (96). This oxidative burst is crucial for NET formation. Inflammatory cytokines and free fatty acids hinder insulin signaling, activating inflammatory pathway mediators such as IKKβ and JNK1. This results in the translocation of NFκβ to the nucleus, triggering the activation of proinflammatory genes necessary for priming. Additionally, high extracellular glucose promotes a proinflammatory M1 phenotype in macrophages. The interaction between NETs and M1 macrophages exacerbates the proinflammatory response, leading to apoptosis and the release of extracellular DNA. This NET-mediated process contributes to an increased burden of free DNA and disease progression in DN. Understanding the complex pathomechanisms of DN highlights the notable role of NETosis, presenting an opportunity to target NETosis as an emerging therapeutic approach for DN.

### Natural products and therapeutic approaches

Natural products have gained increasing attention as potential therapeutic agents for DN. Recent studies (97–99) have identified comprehensive therapeutic approaches, including natural products, that may provide potential treatment strategies for DN. One example of a natural product with therapeutic potential is colchicine. A recent clinical (100) investigation explored the beneficial effects of low-dose colchicine on neutrophil-related chronic inflammation in DN patients. The findings demonstrated that low-dose colchicine effectively and safely attenuated neutrophil-related chronic inflammation in DN patients with concomitant microalbuminuria in type 2 diabetes. Plant-derived compounds, such as resveratrol (101), curcumin (102), and quercetin (103, 104), have exhibited anti-inflammatory, antioxidant, and renoprotective effects, potentially mitigating the development and progression of DN. Further research is needed to elucidate the underlying mechanisms of action and optimize the therapeutic potential of these natural products. This includes investigating their effects on key molecular pathways involved in DN, such as oxidative stress, inflammation, apoptosis, and fibrosis. Additionally, studies exploring the synergistic effects of natural product combinations or their interaction with conventional therapies could provide valuable insights into their clinical utility.

### Timely focus on the risk of DN complications

Studies (105, 106) have shown a higher risk of urinary tract infections (UTIs) events in individuals with DN, supported by ample epidemiological evidence. Regional studies (107) indicate a UTI prevalence of 25.3% among individuals with diabetes, with females accounting for 41.1% of cases. Elevated blood glucose levels create a favorable environment for bacterial growth, increasing susceptibility to infection by promoting colonization of uropathogens and compromising the immune system. Untreated UTIs in T2DM are closely associated with the risk of DN. They can worsen the pro-inflammatory state by releasing cytokines and inflammatory mediators that damage the kidneys and promote DN progression (108). Numerous studies (109, 110) have explored the potential of uromodulin (UMOD) in preventing UTIs and preserving kidney function. UMOD, also known as Tamm-Horsfall protein, is predominantly produced by cells in the kidney’s thick ascending limb of the loop of Henle (111). It is the most abundant protein in urine and exhibits antibacterial properties. UMOD is believed to play a vital role in UTI defense by acting as a barrier. It binds to bacterial pathogens in the urinary tract, impeding their adherence to the urothelium and subsequent colonization (110). This mechanism reduces the risk of UTIs and restricts infection spread to the kidneys. Given UMOD’s antibacterial effects and potential protective role, it presents an appealing therapeutic target for preventing UTIs and preserving kidney function in individuals with DN. Future research can focus on elucidating the underlying mechanisms of UMOD in UTI prevention, exploring its potential as a diagnostic or prognostic marker for UTIs in DN, and developing interventions to enhance UMOD expression or function. In addition, since UTIs are associated with the possibility of affecting fetal development (112), the resulting targeting of content related to urinary tract infections emphasizes attention to the risk of DN complications.

## Conclusion

In conclusion, DN often co-occurs with cardiovascular complications including hypertension, coronary artery disease and cerebrovascular diseases (113). A comprehensive approach integrating the management of these comorbidities is imperative. Tackling the global vascular burden may lower mortality and enhance quality of life. Future research should further elucidate DN pathogenesis and discover novel treatment paradigms. Targeting inflammation, oxidative stress and vascular dysfunction with drugs and interventions represents major opportunities. Advancing individualized and precision approaches through technological evolution will transform DN care. Promoting an integrated vascular risk reduction strategy also benefits long-term outcomes. In summary, DN management is evolving towards personalized, precise, holistic models. Novel mechanism-based therapies, especially those targeting pathogenic pathways, combined with individualized/precision regimens and comprehensive comorbidity control, will optimize therapeutic strategies for better patient outcomes.

## Data availability statement

The original contributions presented in the study are included in the article/supplementary material. Further inquiries can be directed to the corresponding authors.

## Author contributions

XQZ: Conceptualization, Formal Analysis, Writing – original draft, Writing – review & editing. JZ: Conceptualization, Formal Analysis, Writing – original draft, Writing – review & editing. YR: Formal Analysis, Writing – original draft, Writing – review & editing. RS: Conceptualization, Methodology, Writing – original draft, Writing – review & editing. XZ: Conceptualization, Funding acquisition, Writing – original draft, Writing – review & editing.
